# Identification of potential cancer-related pseudogenes in lung adenocarcinoma based on ceRNA hypothesis

**DOI:** 10.18632/oncotarget.19933

**Published:** 2017-08-04

**Authors:** Yunzhen Wei, Zhiqiang Chang, Cheng Wu, Yinling Zhu, Kun Li, Yan Xu

**Affiliations:** ^1^ College of Bioinformatics Science and Technology, Harbin Medical University, Harbin 150081, China

**Keywords:** pseudogenes, ceRNA, lung adenocarcinoma

## Abstract

Pseudogenes are initially regarded as non-functional genomic fossils resulted from inactivating gene mutations during evolution. Far from being silent, pseudogenes are proved to regulate the expression of protein-coding genes through function as microRNA sponge *in vivo*. The aim of our study was to propose an integrative systems biology approach to identify disease pseudogenes base on competitive endogenous RNA (ceRNA) hypothesis. Here, we applied our method to lung adenocarcinoma (LUAD) RNASeq data from TCGA and identified 33 candidate pseudogenes. We described the characteristics of the candidate pseudogenes and performed functional enrichment. Through analyzing neighboring genes we found these pseudogenes were surrounded by tumor genes and may involve in tumor pathway. Furthermore, the DNA methylation analysis indicated that 21 pseudogenes co-methylated with their competitive mRNAs. In the co-methylated network, we discovered 6 differentially expressed pseudogenes, which we termed potential LUAD-associated pseudogenes. We further revealed that the 3 ceRNA triples (miR-21-5p-NKAPP1-PRDM11, miR-29c-3p-MSTO2P-EZH2 and miR-29c-3p-RPLP0P2-EZH2), whose high risk groups were associated with the poor prognosis of LUAD, may be considered as potential prognostic signatures. Moreover, by integrating target information of microRNA we also provided a new perspective for the discovery of potential small molecule drugs. This work may facilitate cancer research and serve as the basis for future efforts to understand the role of pseudogenes, develop novel biomarkers and improve knowledge of tumor biology.

## INTRODUCTION

Pseudogenes, a sub-class of long non-coding RNAs (lncRNAs) that developed from protein-coding genes (PCGs) but have lost the ability to produce proteins, have long been described as non-functional genomic relicts of evolution [1]. However, it is becoming clear that some of pseudogenes have important regulatory roles in cells. Far from being silent, pseudogenes participate in various biological activities, including being a part in the transcription process [2], or participating in the formation of small interfering RNA (siRNA) which regulated gene expression through RNA-interference pathway [3, 4]. Several studies also implicate dysregulation of pseudogenes as contributing factor in human cancer, with early example such as KRASIP [5].

Notably, an increasing number of studies describe pseudogenes that act as critical effectors in cancer progression [6]. For example, NANOG and OCT4 are essential transcription factors for the maintenance of pluripotency in embryonic stem cells [7, 8], while their pseudogenes, NANOGP1 and POU5F1P1, are aberrantly expressed in human cancers [9]. Poliseno *et al*. had shown that the pseudogene PTENP1 regulated the expression of tumor suppressor PTEN through binding microRNA and took part in tumor biological processes [10]. More recently, Florian *et al*. had provided an evidence that the BRAF pseudogene acted as a competitive endogenous RNA (ceRNA) and induced lymphoma *in vivo* [11]. These studies provide key insights into the potential role of pseudogenes in tumor biology. While intriguing, all of them are still limited in individual pseudogenes, and it is likely that more pseudogenes have roles in oncogenic programs. Therefore, it is essential to perform a systematic analysis across large patient sample cohorts to identify cancer-related pseudogenes. The idea was first explored in 13 cancer using RNA-Seq resource of 293 samples, revealing associations between pseudogene expression and cancer progression [12]. However, though pseudogenes has been reported to act as microRNA sponges that compete with mRNAs to attract microRNAs for interactions and influence the expression of mRNAs [13], the biological characteristic and clinical relevance of pseudogenes that function as ceRNAs remain unclear.

In order to systematically describe cancer-related pseudogenes that act as ceRNAs, here, compared with previous studies that identified ceRNA pairs [14–16], we developed a computational framework and gradually identified LUAD-related pseudogenes. We first obtained the RNA-seq transcript data of LUAD that made available from TCGA and selected positive pseudogene-mRNA interactions based on ceRNA hypothesis. These candidate pseudogenes were characterized in several ways, including transcript length, exon numbers, evolutionary conservation, neighboring gene analysis and co-methylation analysis. Then we inferred potential prognostic biomarker and small molecule drugs for LUAD treatment. Taken together, our study systematically characterized pseudogenes, provided a foundation for deeper understanding the role of LUAD-related pseudogenes and improved knowledge of tumor biology.

## RESULTS

### Identification of LUAD-related candidate pseudogenes that function as ceRNA

**Figure 1:**
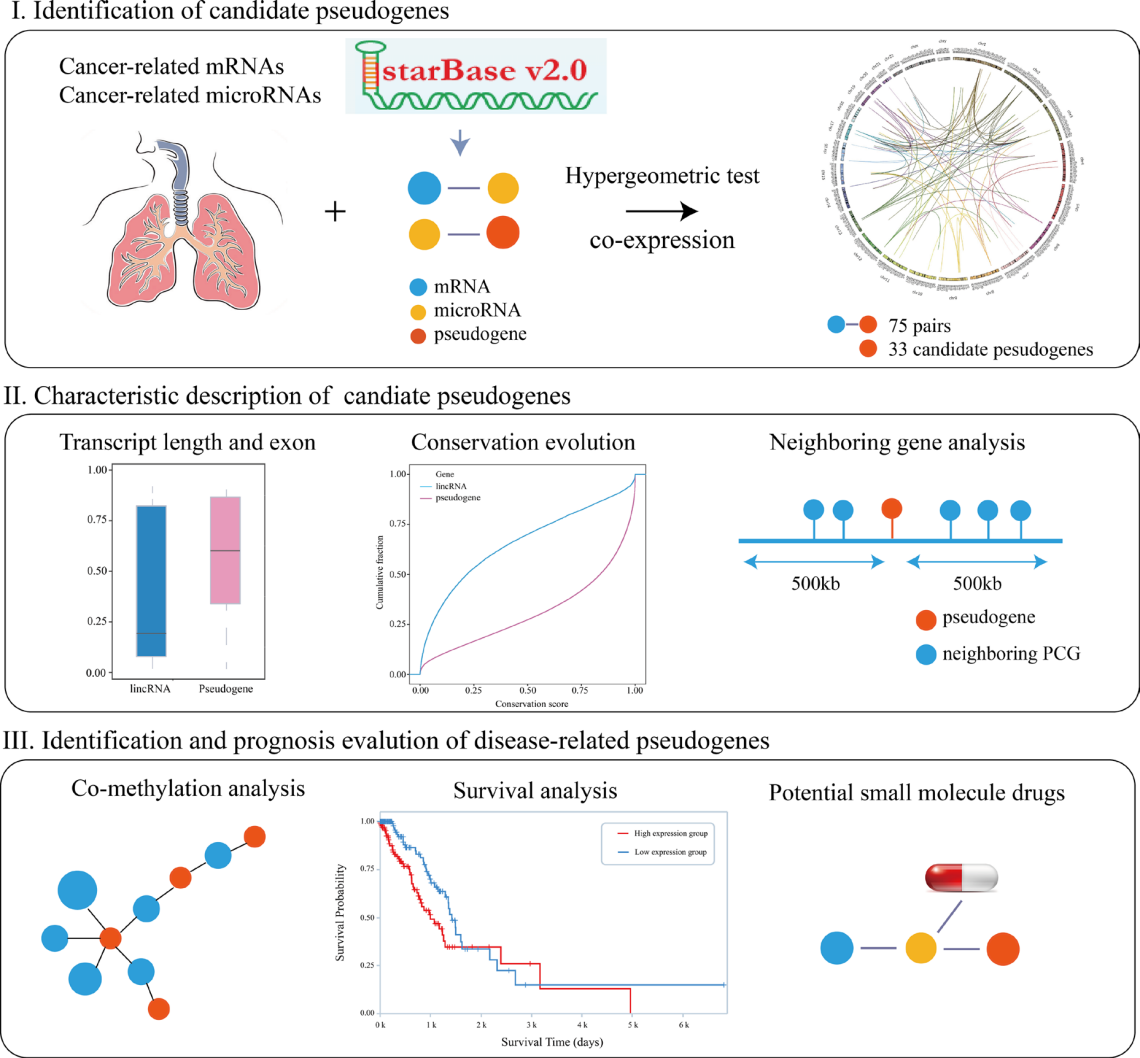
Work flow

We constructed a framework to identify and analysis disease pseudogenes (Figure 1). First, we proposed a pipeline to gradually identify significant pseudogene-microRNA-mRNA triples. After processing the RNASeqV2 data of 576 LUAD samples, we obtained 729 pseudogenes and 16,610 mRNAs profiles respectively. Furthermore, based on the target information, we obtained 434,691 pseudogene-microRNA-mRNA triples. The mRNAs and microRNAs in those triples were LUAD-related which were selected from cancer databases. Recent studies had shown that the two microRNA sponges were more correlative with each other if they shared more microRNAs [14]. In order to identify candidate pseudogene-mRNA competing pairs, a hypergeometric test was used to compute the significance of shared microRNAs for each possible gene pair. All *p*-values were subject to *FDR* correction and 750 pseudogene-mRNA pairs with *FDR* < 0.05 were remained for further analysis. Moreover, in order to reduce the false positive rate of result, all the candidate pseudogene-mRNA pairs with Pearson Correlation Coefficient (*PCC)* ≥ 0.259 and *p*-adjusted < 0.05 were identified as ceRNA-ceRNA interactions. In total, 33 pseudogenes were identified as candidate LUAD-related genes (Supplementary Data 1). In addition, we found the mRNAs in candidate ceRNA pairs were enriched in several critical pathways, such as *Jak-STAT signaling pathway*, *Adipocytokine signaling pathway*, *MicroRNA in cancer* and *One carbon pool by folate*. These observations suggested that some of the candidate pseudogenes may be members of those signaling pathways and prompt cancer development.

### Properties of candidate pseudogenes

**Figure 2:**
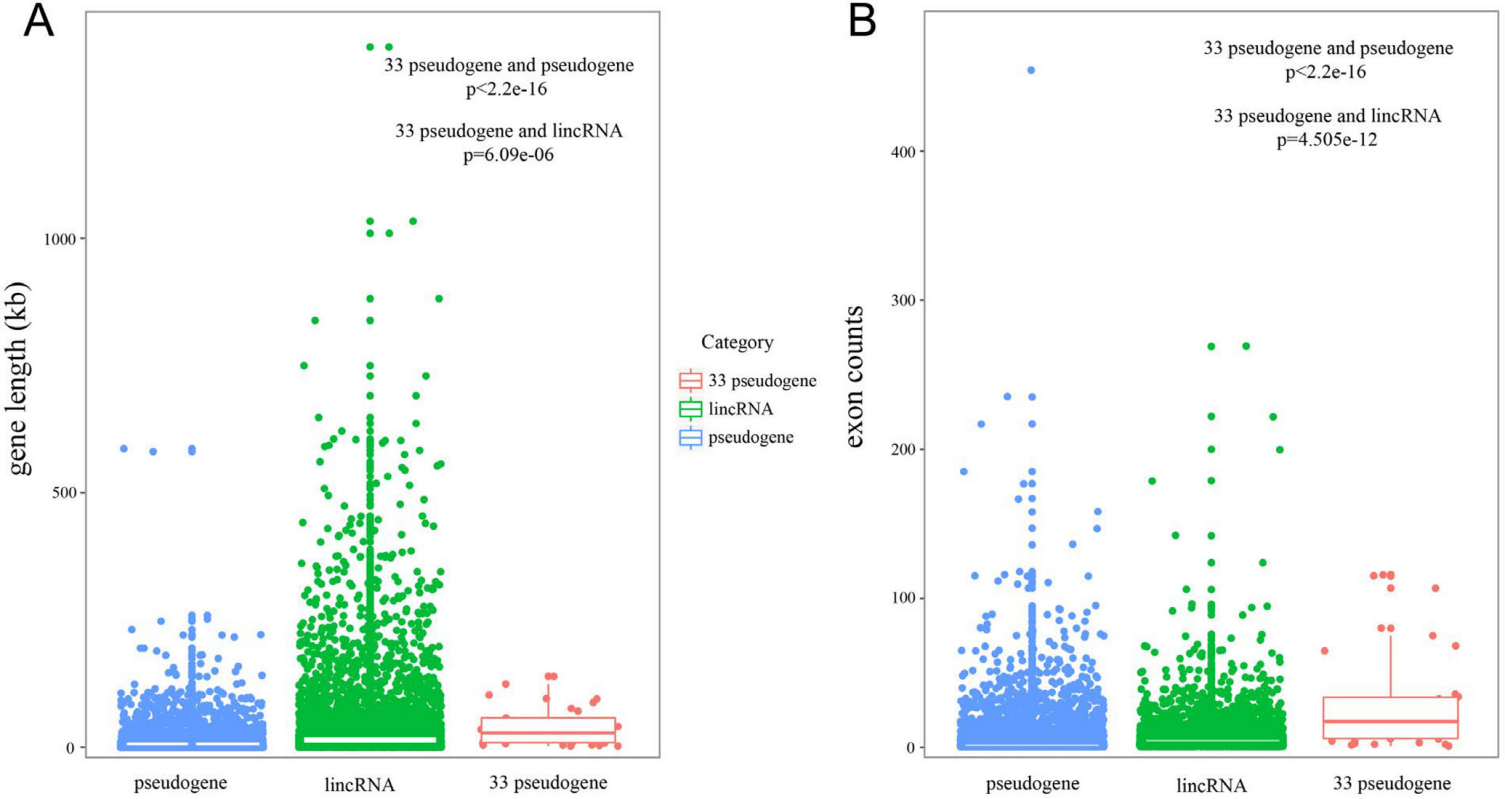
The properties of candidate pseudogenes (**A** and **B**) The boxplot depicted the transcript length and exon number of genes respectively.

We explored the transcript length and exon number of candidate pseudogenes, and compared these properties with all pseudogenes and lincRNAs. Transcripts for candidate pseudogenes were longer than those for all pseudogenes and lincRNAs were (Figure 2A, candidate pseudogenes versus all pseudogenes, *p* < 2.2e-16; candidate pseudogenes versus lincRNAs, *p* = 6.09e-06). Moreover, candidate pseudogenes had more exons per transcript than all pseudogenes and lincRNAs did (Figure 2B, candidate pseudogenes versus all pseudogenes, *p* < 2.2e-16; candidate pseudogenes versus lincRNAs, *p* = 4.505e-12). Previous study pointed out that the lncRNAs with long transcripts and a large number of exons may be involved in the function as microRNA sponges in biological processes [17, 18]. These results suggested that the candidate pseudogenes may also function as ceRNA and have key functions in cancer.

### Candidate pseudogenes had high evolutionary conservation

**Figure 3:**
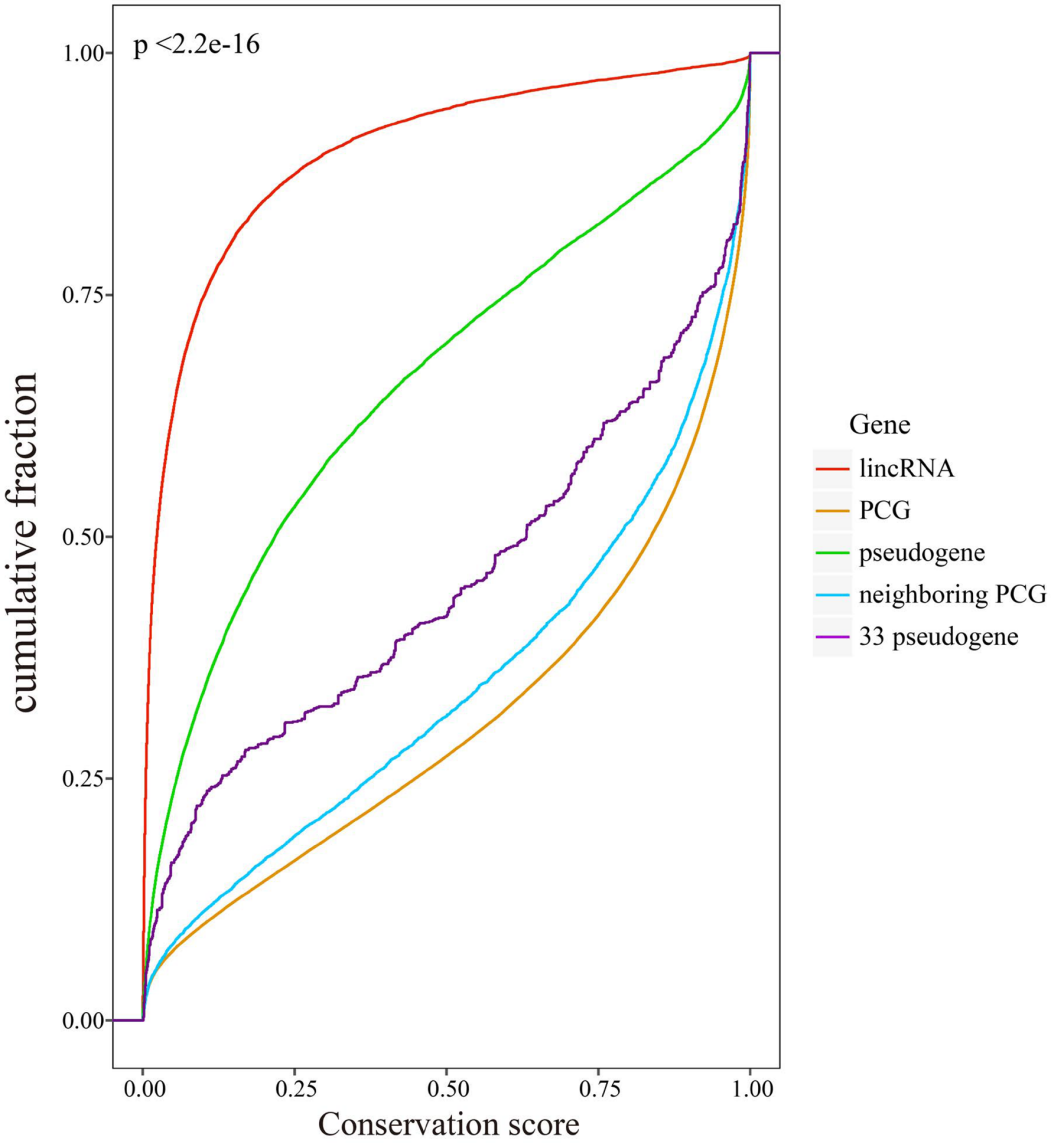
Evolutionary conservation analysis Cumulative distribution of conservation scores of lincRNAs, pseudogenes, PCGs, candidate pseudogenes and neighboring PCGs.

Next, we evaluated the evolutionary conservation of candidate pseudogenes, all pseudogenes, lincRNAs, PCGs and neighboring PCGs respectively by using phastCons scores. The flanking PCGs within 500kb of pseudogenes were considered as neighboring PCGs. Our results showed that pseudogenes and lincRNAs exhibited poor evolutionary conservation relative to PCGs (Figure 3). We also found that all pseudogenes exhibited relatively higher evolutionary conservation than lincRNAs did (*p* < 2.2e-16). It can be explained by the fact that most intergenic transcripts show little or no evolutionary conservation [19]. Especially, candidate pseudogenes had a remarkably higher conservation than all pseudogenes did (*p* < 2.2e-16), perhaps because of its relatively high percentage of exons. The results indicated that the ceRNA pseudogenes may have important functions in biological process. Therefore, though losing the ability to produce proteins, it was still valuable to analyze the ceRNA pseudogenes.

### Neighboring PCGs analysis

**Figure 4:**
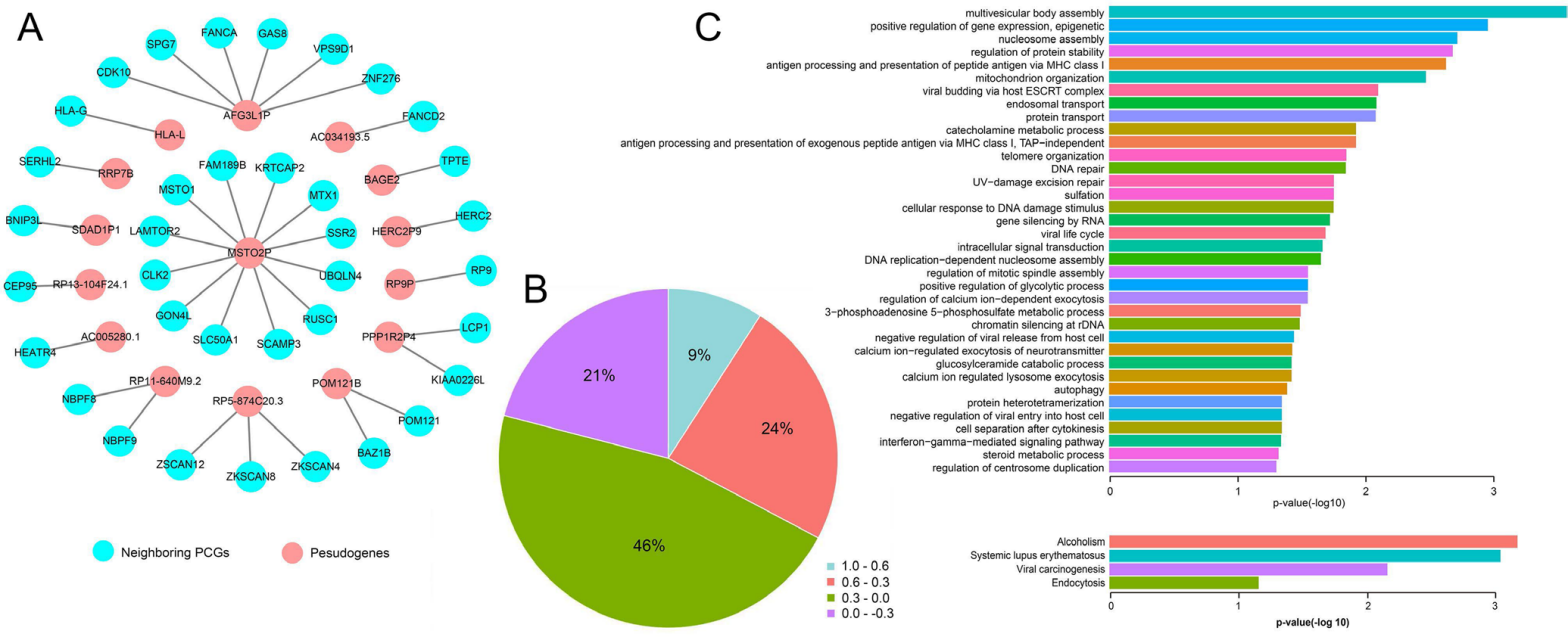
Analysis of neighboring PCGs (**A**) Cis-acting network (*PCC* ≥ 0.6). (**B**) Pie chart. The proportion of *PCC* value between pseudogenes and their neighboring genes. (**C**) GO enrichment and KEGG pathways (*p* < 0.05, *FDR* < 0.05).

We next evaluated the potential roles of candidate pseudogenes in cancer. We calculated the *PCC* value between the candidate pseudogenes and their neighboring PCGs (Figure 4A and 4B). In network, the candidate pseudogenes were surrounded by disease-related genes. For example, MSTO2P was surrounded by 12 genes. LAMTOR2 complex regulated focal adhesion dynamics during cell migration [20] and CLK2 was proved to change in Alzheimer's disease [21]. Furthermore, DAVID v6.8 was used to perform gene ontology (GO) terms and pathway enrichment for neighboring PCGs. The GO semantic annotation showed that neighboring PCGs were enriched in some functions that was related with development and progression of tumors such as *autophagy* and *antigen processing* (Figure 4C). The pathway enrichment of neighboring PCGs also revealed that they participated in cancer-related biology pathway such as *viral carcinogenesis*. The *viral carcinogenesis* pathway revealed the molecular mechanisms and the etiology of human disease [22], which may suggest that candidate pseudogenes were members of these pathway and affected the development and progression of cancer.

### Construction of co-methylation network

**Figure 5:**
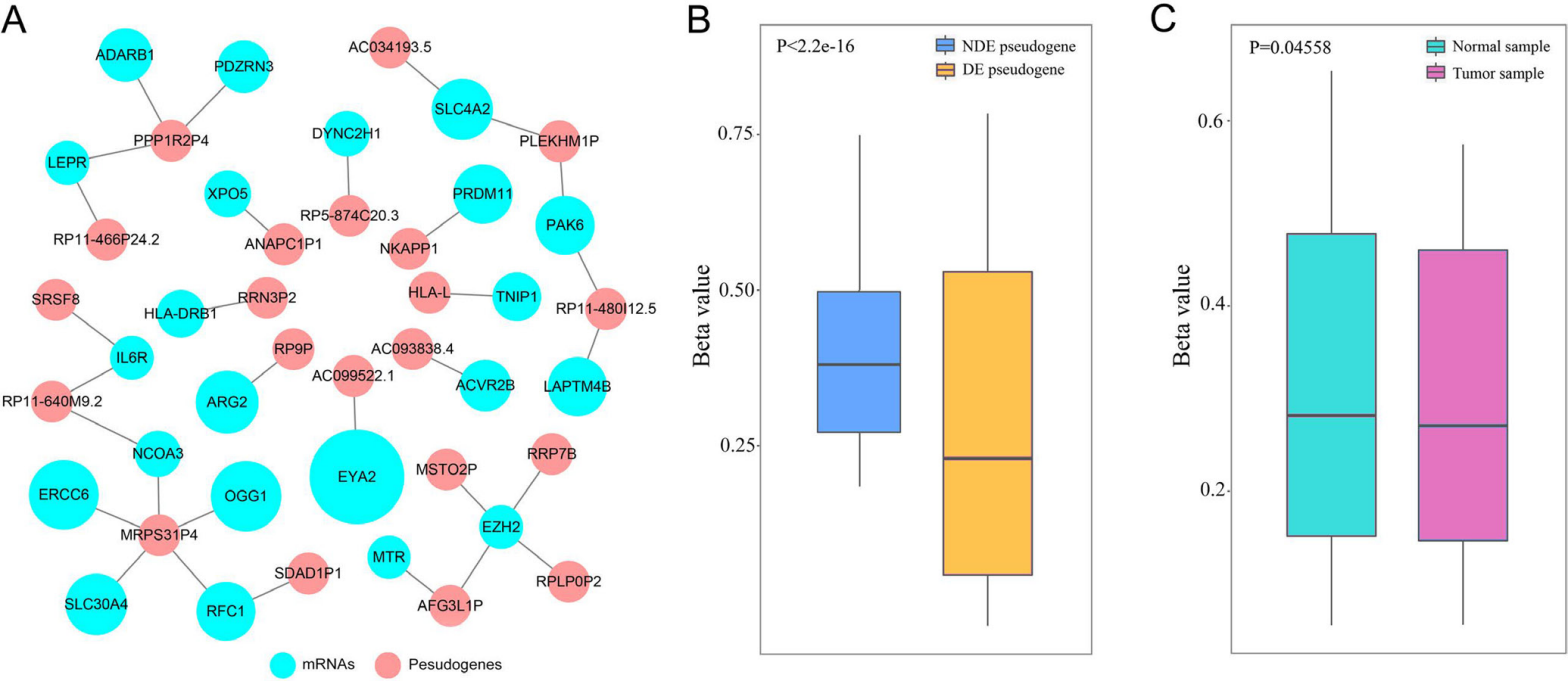
Co-methylation analysis (**A**) Co-methylated network. (**B**) The boxplot depicted the methylation level between DE and NDE pseudogenes. (**C**) The boxplot depicted the methylation level between normal samples and tumor samples (7 differentially expressed pseudogenes).

Previous studies had demonstrated that DNA co-methylation suggested functional associations between gene pairs in cancers [23]. To further explore the relationship between candidate pseudogenes and LUAD-related mRNAs, we performed co-methylation analysis (Figure 5A). The co-methylated network contained 21 pseudogenes and 22 mRNAs (*p* < 0.05, *PCC* ≥ 0.2), and it may reveal the functional association between pseudogenes and their target mRNAs. For example, altered expression of EZH2 was proved to be associated with the risk of lung cancer [24]. In network, 4 pseudogenes (RRP7B, RPLP0P2, MSTO2P and AFG3L1P) co-methylated with EZH2, suggesting an involvement in the progression of lung cancer. Pak6 protein kinase is a novel effector of an atypical Rho family GTPase Chp/RhoV [25]and it co-methylated with pseudogene PLEKHM1P and RP11-480I12.5, indicating that the two pseudogenes may take part in cancer biological processes. In addition, 6 of the 21 pseudogenes were differentially-expressed (DE) genes (Supplementary Data 2). We further analyzed DNA methylation pattern of the 6 DE pseudogenes. The result showed that the methylation level of DE pseudogenes was lower than that of non-differentially expressed (NDE) pseudogenes (Figure 5B). Accumulating evidences indicated that hypomethylation was an important phenomenal characteristic. For example, a study proved that body-hypomethylated human genes were prone to cancer-associated dysregulation [26]. Therefore, it can be inferred that the DE pseudogenes played key roles in cancer processes and we selected them for further analysis. We also explored the methylation pattern of DE pseudogenes in tumor samples and normal samples, and the methylation level in normal samples was higher than that of tumor samples (Figure 5C).

### Prognostic ceRNAs in LUAD

**Figure 6:**
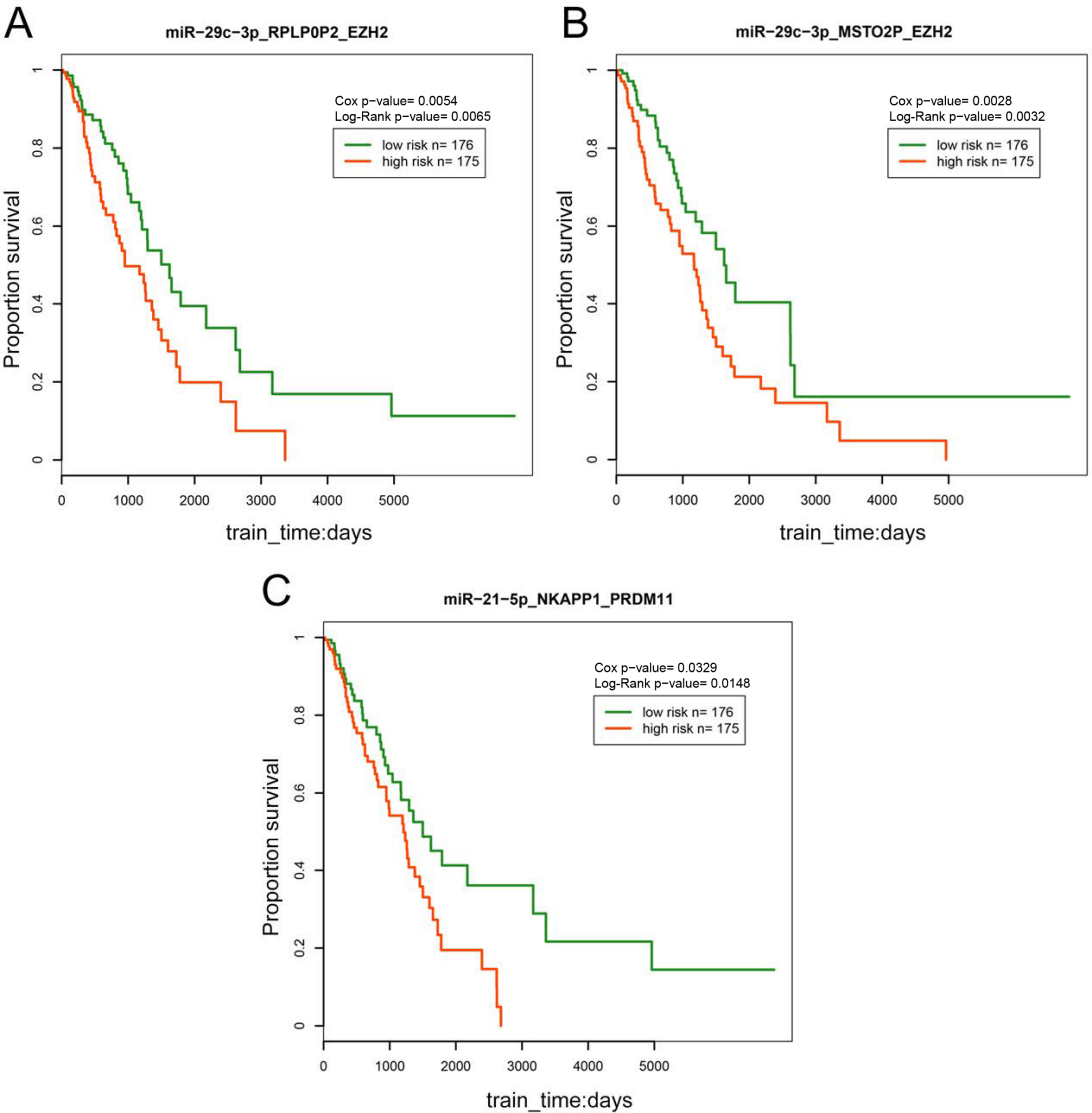
Survival analysis of the ceRNA triples

To explore the influence of the ceRNA triples on patient survival, we calculated the risk score for each pseudogene-microRNA-mRNA triple based on risk score of single factor regression analysis of each node, and divided patient samples into high-risk and low-risk groups by median, then drew Kaplan-Meier curves with R software. Cox *p*-value was used to evaluate the significance between expression of triples and overall survival. Log-Rank test *p*-value was used to test the significance between the two groups of patient samples. The result exhibited that the 3 triples (miR-21-5p-NKAPP1-PRDM11, miR-29c-3p-MSTO2P-EZH2 and miR-29c-3p-RPLP0P2-EZH2) were significantly associated with prognosis (*p* < 0.05, Figure 6). The high-risk group consisting of patients with high risk scores had lower survival time, which revealed that the high risk group was associated with the poor prognosis of LUAD. In addition, RPLP0P2 was proved to be associated with cell proliferation and adhesion in LUAD tumor cells [27]. It was suggested that the patient survival could be affected by the ceRNA pairs. These results indicated that the 3 ceRNA triples may serve as potential biomarkers of LUAD and contribute to the following treatment.

### Potential small molecule drugs for LUAD treatment

**Figure 7:**
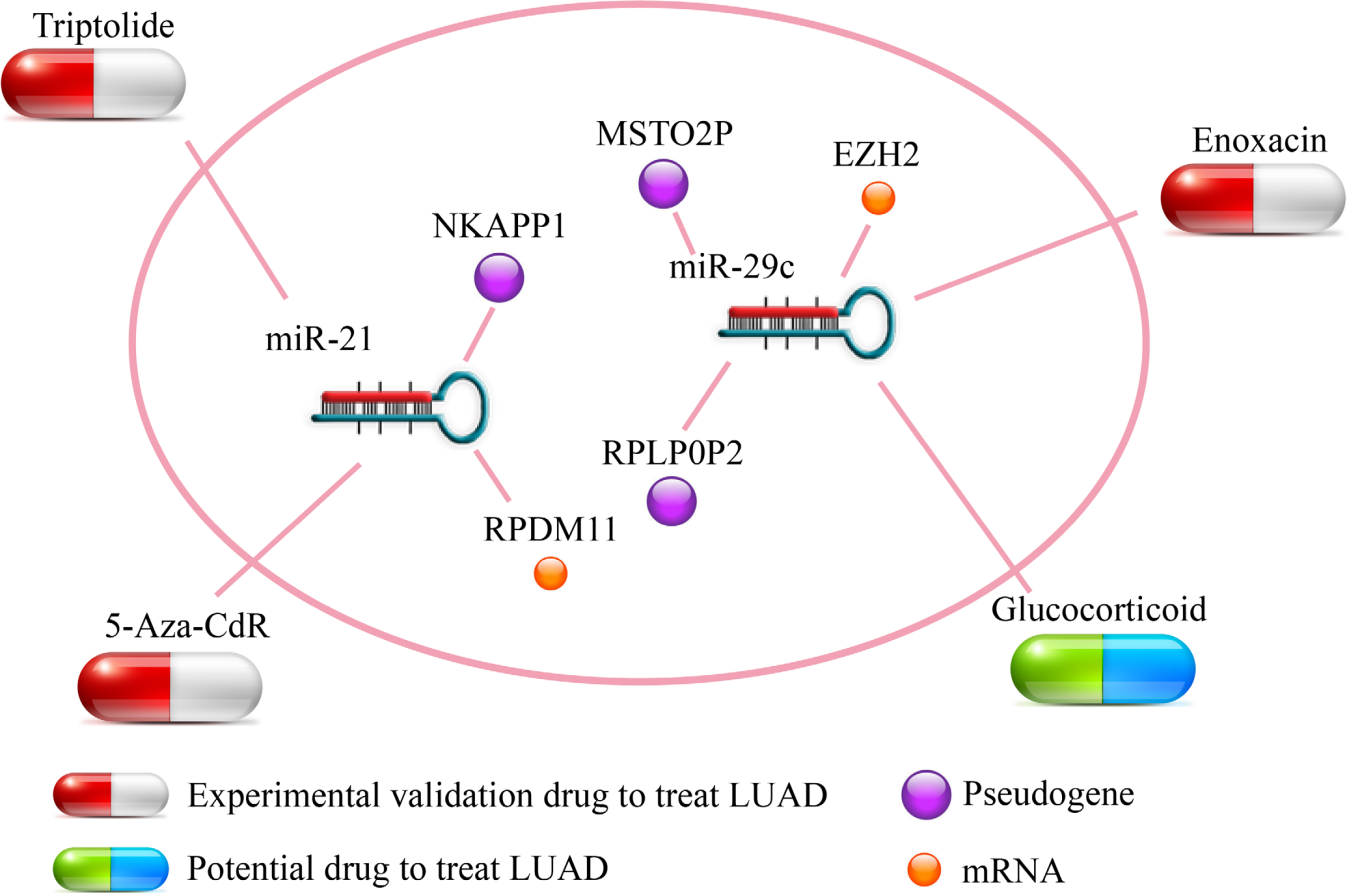
Potential small molecule drugs for LUAD treatment The capsules represented experimental validation drugs and potential drug respectively. The nodes represented pseudogenes and LUAD-related mRNAs respectively.

In the pseudogene-microRNA-mRNA triples, the perturbation of microRNA expression can influence the expression level of pseudogenes and mRNAs. Moreover, several studies had verified that bioactive small molecules could regulated microRNA expression [28, 29]. Here, referring to a previous work [30], we combined the information that provided by SM2miR [31] with the module to infer potential small molecule drugs for LUAD treatment. In the triples, some of the potential drugs could up/down-regulate the microRNA expression and further down/up-regulate the expression of pseudogenes and contribute to the treatment of LUAD (The risk coefficient of miR-21was 0.382, so that we needed to down-regulate it; the risk coefficients of miR-29 were -0.303 and -0.381 respectively, thus we needed to up-regulate them). In miR-21-5p-NKAPP1-PRDM11, 5-aza-2’-deoxycytidine (5-Aza-CdR) could down-regulate the miR-21, and up-regulate the expression of corresponding pseudogene/mRNA. It was proved to be a potent inhibitor of DNA methylation for therapy of advanced non-small cell lung cancer [32]. Triptolide, a natural diterpenoid compound, was proved to be an inhibitor of lung inflammation. In addition, for miR-29c-3p-RPLP0P2-EZH2/miR-29c-3p-MSTO2P-EZH2 triples, Enoxacin can inhibit RNA helicase DHX9 in lung cancer and was an effective agent for lung cancer prevention and treatment [33]. Glucocorticoid could also up-regulate the expression of miR-29c and involve in important biological pathway [34] while it was not mentioned in LUAD. Therefore, we inferred that those small molecule drugs which regulated the expression of microRNAs in ceRNA triples, may serve as potential drugs for LUAD treatment (Figure 7 and Supplementary Data 3).

## DISCUSSION

Recently, pseudogenes have emerged as new players in tumor biology. However, the way of effectively identifying cancer-related pseudogenes that functions as ceRNA still remains unclear. Here, by establishing a novel computational framework we systematically explored the potential role of pseudogenes as microRNA sponges for LUAD. Notably, to ensure the accuracy of prediction power, positive pseudogene-mRNA interactions was identified by strict criteria: (i) both of the mRNA and microRNA in each triple must be verified by disease databases; (ii) each possible mRNA-pseudogene pair must significantly share common microRNAs which can interact with both of them (hypergeometric test, *FDR* < 0.05); (iii) only top correlated mRNA-pseudogene pairs, whose correlation coefficients were higher than the threshold of the 90th percentile of the corresponding overall correlation distribution (*PCC* ≥ 0.259), were regarded as candidate pseudogene-mRNA interactions. Moreover, DE analysis that has been used frequently in mRNA and microRNA research may not be suitable for identifying microRNA sponge interactions [35]. The reason is that the pseudogenes that are not DE may affect the amount of the microRNAs available for bindings [36]. Therefore, the DE analysis between normal samples and tumor samples of pseudogenes was performed after getting candidate pseudogenes. The candidate pseudogenes had relatively long transcripts and plenty of exons, suggesting they may have important functions. In addition, we observed that candidate pseudogenes had high evolutionary conservation, while previous studies had revealed that the evolutionary conservation of lncRNAs was poor [37]. It may suggest that ceRNA pseudogenes had highly evolutionary conservation and played important roles in biological progress. Although the existing classification of lncRNAs still faced with challenge [38], the above analysis suggested that pseudogenes may serve as an independent category because of the unique characteristic. Through neighboring PCGs analysis, we found candidate genes were surrounded by oncogenes, and they may be members of cancer pathways. Importantly, utilizing 450k DNA methylation data from TCGA, we found 21 pseudogenes co-methylated with mRNAs and 6 of them were differentially-expressed. We showed different methylation levels between DE and NDE pseudogenes in gene-body regions, which suggested methylation level may affect the expression of DE pseudogenes [39]. The 6 differentially-expressed pseudogenes co-expressed and co-methylated with experiment-validated LUAD-related mRNAs. Hence, we inferred they were potential LUAD-related pseudogenes. To infer clinical relevance of the LUAD-related pseudogenes, survival analysis was also taken into consideration. We found that the pseudogene RPLP0P2, one of the 6 potential LUAD-related genes, was reported to be associated with decreased cell proliferation and adhesion ability in LUAD [27, 40]. These analyses revealed that the 6 pseudogenes may be functional involved in tumorigenesis and implied a novel prognostic strategy for cancer treatment. At last, we provided a new perspective for the discovery of potential small molecule drugs and expected to find effective drugs for cancer treatment in the future.

In recent years, a growing body of studies has focused on functional pseudogenes that played critical roles in human diseases. For example, Zheng *et al*. analyzed the pseudogene CYP4Z2P based on ceRNA hypothesis in breast cancer [41]. However, cancer-related pseudogenes may affect many genes in ceRNA network while they solely focus on single pseudogene or gene. Although the methods were all based on ceRNA hypothesis, our study had two advantages over them: (i) a strict computational pipeline was applied to identify candidate ceRNA pairs; (ii) the identified pseudogenes were differentially-expressed and co-expressed/methylated with experiment-verified LUAD-related gene. Certainly, as with any computational approach, our framework was limited by the quality and quantity of the input data. Further power is anticipated by including additional samples, complement of methylation probes, and better interaction networks. For example, since strict criteria were used in our study, few datasets was suitable for identifying cancer-related pseudogenes, which implies that our framework could be more accurate by the complement of data in the future.

In summary, we provided a framework to identify cancer-related pseudogenes and integrate them with genomic analysis. These candidate cancer-related pseudogenes could be further evaluated as potential therapeutic targets.

## MATERIALS AND METHODS

### Expression profiles of pseudogene and mRNA in LUAD

The RNA-seq V2 data of LUAD patient samples was obtained from TCGA project (http://cancergenome.nih.gov/) [42], including 517 tumor samples and 59 adjacent normal samples (Supplementary Table 1). GENCODE hg19 genome was used as a reference. The reads were mapped to the exons of mRNAs and pseudogenes. The pseudogenes/mRNAs that overlapped with mRNAs/pseudogenes were excluded. RPKM value was calculated to evaluate the expression levels of pseudogene and mRNA:

$$\text{RPKM}=10^{9}\frac{C}{NL}$$

Where *C* is the number of mapped reads for pseudogene or mRNA, *N* is the number of total mapped reads, *L* is the length of the pseudogene or mRNA. To reduce false positive rate, pseudogenes or mRNAs with missing values in > 50% of the sample were removed [14]. Next, we added 0.00001 to the expression value of each gene and performed log2-transformed. In total, we obtained 729 pseudogenes and 16,610 mRNAs for further analysis.

### Argonaute CLIP-supported microRNA-target interactions

Recently, several studies have reported that the use of cross-linking and Argonaute (Ago) immunoprecipitation coupled with high-throughput sequencing (CLIP-Seq) could identify endogenous genome-wide interaction maps for microRNAs [43, 44]. To investigate human microRNA-target regulatory relationships, the Human microRNA-mRNA interactions were collected from five prediction programs including TargetScan [45], PicTar [46], PITA [47], miRanda [48] and RNA22 [49] in starBase v2.0 [50]. By integrating the above databases, a total of 423,405 non-redundant microRNA-mRNA interactions were used in our study. The microRNA-pseudogene interactions were also collected from starBase v2.0, including 16,126 interactions pairs.

### Collection of LUAD-associated mRNAs and microRNAs

Several database systems have proposed to provide a comprehensive resource of mRNAs and microRNAs dysregulation in various human diseases. LUAD-related mRNAs were collected from four databases, including COSMIC [51], OMIM [52], GAD [53] and Phenopedia-Genopedia database [54]. In addition, experimentally verified LUAD-related microRNAs were obtained from HMDD [55], miR2Diease [56], miREnvironment [57] and OncomiRDB [58].

### Identification of potential LUAD-related pseudogenes

Having got the Ago CLIP-supported mRNA-microRNA and pseudogene-microRNA regulatory data, we performed a three-step pipeline to gradually identify LUAD-related pseudogenes that acted as microRNA sponges based on the ceRNA hypothesis. First, the pseudogene-microRNA-mRNA triples were obtained using predicted microRNA target information, in which all of the microRNAs and mRNAs were selected by disease database. Second, in order to identify competing pseudogene-mRNA interactions, a hypergeometric test was performed to evaluate the significance of shared microRNAs for each possible gene pair:

$$\text{P}=1-\sum_{i=0}^{x-1}\frac{\left(\begin{array}{c} L\\ i \end{array})(\begin{array}{c} N-L\\ M-i \end{array}\right)}{\left(\begin{array}{c} N\\ M \end{array}\right)}$$

Where *N* is the total number of microRNAs which were associated with pseudogene or mRNA, *M* is the number of microRNAs interacting with this given pseudogene, *L* is the number of microRNAs interacting with this given mRNA, and *x* is the number of microRNAs that interact with both of them, respectively. The *p*-value and *FDR* correction less than 0.05 were used as the threshold [59]. Finally, in order to reduce the false positive rate of result, the pseudogene-mRNA pairs that *P* < 0.05, *PCC* ≥ 0.259 (75 pairs, 10% top correlated pseudogene-mRNA pairs, including 33 pseudogenes and 40 mRNAs) were considered to be potential pseudogene-mRNA interactions [16].

### Evolutionary conservation analysis

We evaluate the evolutionary conservation of all pseudogenes, lincRNAs, PCGs, candidate pseudogenes and their neighboring PCGs. The evolutionary conservation was evaluated by 46-way phastCons vertebrate conserved elements from the UCSC Genome Browser website [60]. We considered a base as a unit and computed average phastCons scores for exons.

### DNA methylation analysis

DNA methylation data of LUAD from Illumina Infinium Human Methylation 450 Beadchip (Infinium 450 k) arrays was obtained from TCGA, including 485,577 probes, 475 tumor samples and 32 normal samples (Supplementary Table 1). We then assigned the probes into the gene-body regions.

To estimate the methylation level of a given probe, we used the beta value: the ratio of intensities between methylated and unmethylated alleles. The beta value was obtained from the level 3 Infinium 450k data in TCGA; the corresponding *p*-value of each probe was obtained from level 2 Infinium 450k data. We only used the beta values with significant detection *p*-values (*p* < 0.05) in calculations to avoid using the missing data [61]. The average value of probes within a gene was regarded as methylated value.

### Survival analysis

The clinical information of LUAD patient samples was obtained from TCGA. Cox regression analysis was used to evaluate the correlation between survival time and pseudogene expression. The risk ratio was used to calculate the risk score for each sample. Then these sample were divided into high-risk and low-risk group based on the mid-value of risk score [62]. The Kaplan-Meier survival method was used to evaluate the influence of the pseudogene for patient prognosis. The Log-Rank test *p*-value was used to test the significance of correlation between two groups of patients (*p*-value < 0.05). The Cox *p*-value was used to evaluate the significant correlation between the overall survival and genes (*p*-value < 0.05).

### Statistical analyses

The *p*-value and *FDR* correction less than 0.05 were used as the threshold in the hypergeometric test. The pseudogenes that fold change > 1.5 and *FDR* < 0.01 were considered as DE pseudogenes. Functional enrichments of mRNAs were consisted on the Fisher's exact test (two-tailed) implemented by DAVID v6.8 (https://david-d.ncifcrf.gov/) [63]. Wilcox rank sum test was used to test the significance between two groups of data (*p* < 0.05).

## SUPPLEMENTARY MATERIALS TABLES









## Abbreviations

LUAD: lung adenocarcinoma
PCGs: protein-coding genes
ceRNA: competitive endogenous RNA
siRNA: small interfering RNA
DE: differentially expressed
NDE: non-differentially expressed
PCC: pearson correlation coefficient
