# Supplementary material for: Identification of potential cancer-related pseudogenes in lung adenocarcinoma based on ceRNA hypothesis
Source: Oncotarget. 2017 Aug 4;8(35):59036–47. doi: 10.18632/oncotarget.19933 (PMC5601712; doi:10.18632/oncotarget.19933)
Supplement: Supplementary file 1 [file oncotarget-08-59036-s001.pdf]

Identification of potential cancer-related pseudogenes in lung adenocarcinoma based on ceRNA hypothesis

SUPPLEMENTARY MATERIALS

Supplementary Table 1: The description of data

Name	Type	source	Annotation	Tumor sample	Normal sample
mRNA	RNASeqV2	TCGA	hg19	517	59
Pseudogene	RNASeqV2	TCGA	hg19	517	59
Methylation	Infinium 450k	TCGA	hg19	475	32

Supplementary Data 1: 33 candidate pseudogenes. See Supplementary_Data_1

Supplementary Data 2: 6 DE pseudogenes. See Supplementary_Data_2

Supplementary Data 3: Small molecule drugs that up/down-regulated microRNAs. See Supplementary_Data_3
